# The potential therapeutic effects of the gut microbiome manipulation by synbiotic containing-*Lactobacillus plantarum* on neuropsychological performance of diabetic rats

**DOI:** 10.1186/s12967-019-02169-y

**Published:** 2020-01-10

**Authors:** Mohammad Morshedi, Maryam Saghafi-Asl, Elaheh-Sadat Hosseinifard

**Affiliations:** 1 Drug Applied Research Center, Tabriz University of Medical Sciences, Tabriz, Iran; 2 Nutrition Research Center, School of Nutrition and Food Sciences, Tabriz University of Medical Sciences, Tabriz, Iran; 3 Department of Clinical Nutrition, School of Nutrition and Food Sciences, Tabriz University of Medical Sciences, Tabriz, Iran

**Keywords:** Microbiota, Probiotic, Behavior, Dysbiosis, Diabetes, Gut–brain axis

## Abstract

**Background:**

The manipulation of gut microbiota as a target has been suggested to reduce the risks for a number of diseases such as type 2 diabetes mellitus (T2DM). Conversely, T2DM is associated with complications such as gut and brain disorders. Furthermore, the impact of probiotics and prebiotics to improve T2DM complications are reported. Thus, the present study seeks to investigate the therapeutic and neuropsychological effects of *L. plantarum* and inulin in diabetic rats.

**Methods:**

Throughout the investigation, *L. plantarum*, inulin or their combination (synbiotic) was administered to diabetic rats. in the end, fecal samples were collected to evaluate the gut microbial composition. Then behavioral tests were conducted. Subsequently, the obtainment of the prefrontal cortex (PFC) and hippocampal samples.

**Results:**

Our data demonstrated that administration of *L. plantarum* and inulin could improve gut dysbiosis and oxidative stress status. In addition, it could ameliorate serotonin and BDNF/TrkB signaling pathway. Notably, a strong correlation between the gut microbiota changes and cognition responses was observed. Interestingly, synbiotics intake exploited a rather powerful effect on oxidative stress markers.

**Conclusion:**

The findings confirm that there is a beneficial therapeutic potential of supplements, especially symbiotic. Moreover, neuropsychological improvement associated with balanced gut microbiome.

## Background

Type 2 diabetes mellitus (T2DM) is among the one of the most common metabolic diseases which is characterized by hyperglycemia and insulin resistance (IR) [1]. It is captivating to note that it could lead to dysfunctions in various organs of the body such as the heart, kidneys, eyes, and the brain [1–3]. Of equal importance is that the imbalance of the antioxidant system is regarded as one of the main reasons for such complications, which is due to T2DM [2]. Intriguingly, exacerbation of oxidative stress in different regions of the brain can exacerbate the neurological and psychological disorders [3–5]. However, boosting levels of antioxidant enzymes can be convenient to enhance such conditions [6].

Recently, there has been a raise of interest in the gut microbiota as a novel therapeutic target that bridges the gut to the brain [7, 8]. More often than not, the composition of the gut microbiota consists of four main phyla (*Firmicutes*, *Bacteroidetes*, *Actinobacteria* and *Proteobacteria*) where the majority includes the *Firmicutes*. Interestingly enough, there is a controversy regarding the composition and fraction of *Firmicutes* in T2DM. Of interest is that there is suggesting evidence that an unbalanced composition of the gut microbiota (dysbiosis) plays a key role in the alteration of behavioral mechanisms [5, 9, 10]. What’s more, dysbiosis is reported in diabetic patients [11, 12]. Having said that, normalization of the gut microbiota may result in improved central nerves system (CNS) function and brain development [7, 13].

On the other hand, the interaction between the prefrontal cortex (PFC) and hippocampus in relation to cognition has been reported in several studies [14, 15]. Any disruption in the production or function of proteins, neurotransmitters, or their receptors in these two regions can lead to nerve damage and cognitive impairment thanks to the increase in oxidative stress [14, 16, 17]. It is newsworthy to include that brain-derived neurotrophic factor (BDNF) and serotonin are the two synergistic parameters involved in the maintenance of the structure and function of the brain [18]. Following the same subject, brain concentrations of BDNF and serotonin are depressed in T2DM [5]. Previous studies have publicized that damage to the BDNF, tropomyosin receptor kinase B (TrkB), or cyclic adenosine monophosphate responsive element-binding protein (CREB) pathway can result in neurological and behavioral disorders [19]. In view of that, T2DM is a risk factor for cognitive impairment [20].

Synbiotics are beneficial microorganisms (probiotics) plus indigestible food ingredients (prebiotics) that affect the host [5, 21, 22]. Enthrallingly, the administration of either probiotic or prebiotic may contribute to the normalization of dysbiosis alongside improving the complications of T2DM [23]. It is also worth considering that the efficacy of *Lactobacillus* species as a substantial group of probiotics in CNS disorders is still a matter of controversy [24, 25]. Altogether, the present study aimed to investigate the effects of *L. plantarum* and/or inulin intake on gut microbial composition, PFC, and hippocampal concentration of oxidative stress markers, serotonin, BDNF, CREB, and TrkB as well as cognitive function in male diabetic rats.

## Methods

### Animals

A total number of 48 nine-week-old male Wistar rats (250 ± 10 g), obtained from Laboratory Animal Center at Pasteur Institute of Iran, were housed in cages (8 rats per group) at the room temperature of 22–25 ℃ and humidity of 40–60% on an ad libitum typical pellet diet and tap water based on 12-h light/dark conditions. Thereupon, the weight (weekly) and food intake (daily) of the rats were measured. It should also be recalled that the experimental procedures administered on animals were in accordance with the Principles of Laboratory Animal Care guidelines (NIH Publication, revised 1996) as well as the Ethics Committee on Animal Research at Tabriz University of Medical Sciences.

### Experimental design

Following their 1-week adaptation to the mentioned laboratory conditions, the rats were divided on a random basis into 6 groups; i.e. healthy control (Healthy); diabetic Control (D-Control); diabetic + *L. plantarum* (D-Pro); diabetic + inulin (D-Pre); diabetic the *L. plantarum *+ inulin (D-Syn), and diabetic Sham group (D-Sham). Besides, a 4-week high-fat diet was given to these rats which were assumed to be diabetic, prior to the induction of T2DM. In the meantime, the Healthy group received a normal diet (ND). Using a single intraperitoneal (IP) injection of streptozotocin (STZ; Sigma–Aldrich, 35 mg/kg body weight (BW) in a 0.1 mol/L citrate buffer), the T2DM was subsequently induced. Divertingly, with 72 h of STZ injection, the blood sugar (BS) of the T2DM was also reported to be over 250 mg/dL which resulted in the conclusion that the rats were sacrificed for complementary analyses after the 8-week supplementation and subsequent behavioral testing.

### Preparation of supplementation

It is notable to state that the 37 °C, *L. plantarum* ATCC 8014 was cultured in MRS (Man-Rogosa-Sharpe broth, pH 6.8) broth. Using centrifugation, CFU calculated by dilution, and the bacteria were also harvested overnight from MRS broth along with streaking on MRS agar plates at 37 °C. *Lactobacillus plantarum* was thenceforth centrifuged and re-suspended at a dilution of 10^7^ colony-forming units (CFU)/mL in PBS and after that 1 mL gastric gavage was employed for treatment one time a day. Correspondingly, inulin (5% of daily food weight) was dissolved in drinking water.

### Preparation of the blood and tissue samples

Over and above that, 5 mL of the blood sample was obtained from the heart. Still further, the given animals which were anesthetized with pentobarbital sodium IP (Sigma, 65 mg/kg BW) were sacrificed following the behavioral testing, and then the hippocampus and PFC region of their brain tissues were removed at once and on the ice. Each tissue sample was subsequently homogenized and centrifuged at 8000 rpm at 4 °C for 10 min. Lastly, the Bradford test was conducted in addition to determine the total protein concentration of the supernatant [26].

### Biochemical assays

Glutathione peroxidase (GPx), malondialdehyde (MDA), superoxide dismutase (SOD), together with the total antioxidant capacity (TAC) were determined in the hippocampus and PFC region of the brain, as stated by the procedure in previous investigations. Taking all into account, the SOD kit was used to measure SOD activity based on the xanthine–xanthine oxidase Cytochrome C method [27]. Conjointly, the MDA activity measurement was also performed via the analysis of the MDA reaction with thiobarbituric acid (TBA), forming an MDA-TBA adduct [28]. GPx activity was measured through the Paglia-Valentine method in a similar fashion [29], using cumene hydroperoxide as a substrate [30]. Everything considered, serotonin concentrations, and the BDNF in the supernatants were consequently determined by utilizing enzyme-linked immunosorbent assay (ELISA) kits (Shanghai Crystal Day Biotech Co., Ltd.) based on the manufacturer’s protocol.

### Morris water maze test (MWM)

The cognitive function was measured via MWM [31]. In brief, the experiments were fulfilled in a room with fixed extra maze cues. Following that, a circular pool in black was filled with water (25 °C, height of 40 cm). After that, a platform was placed in one of the quadrants. Facing the pool wall, it was possible for the rats to be randomly released into one of the pool quadrants. Finally, time to find the platform to escape was also recorded and analyzed.

### 16S rRNA gene sequence analysis

#### DNA extraction

Using the ZR Fecal DNA Isolation kit, total gDNA was isolated from 150 mg of fecal material (Zymo Research Corporation) based on the manufacturer’s instructions. Be that as it may, the concentrations of double-stranded DNA in the extracts were correspondingly determined by employing the Quant-iT dsDNA Assay Kit and the Qubit fluorometer (Invitrogen).

#### 16S rRNA Gene Amplicon Library preparation and high-throughput sequencing

To continue, the bacterial diversity was examined by pyrosequencing the 464 bp V3–V4 region of the 16S rRNA gene, amplified through the 341F: 5′-CCTACGGGNGGCWGCAG-3′ and 805R: 5′-GACTACHVGGGTATCTAATCC-3′ primers. Afterwards, the PCR amplification was operated using 1× enzyme buffer, 1 U High-Fidelity DNA Polymerase (Fermentas), 0.2 mM dNTPs mixture (Fermentas), 0.5 mM MgCl_2_, 0.5 μM of each of the primers 341F and 805R, and 1–2 μL of the diluted DNA sample. Just the same, the PCR incubation for the bacterial 16S rRNA amplicon library construction was also performed according to the following conditions: 95 °C for 2 min, 30 cycles of 95 °C for 20 s; 56 °C for 30 s; 72 °C for 1 min; and final extension of 72 °C for 5 min. Using the Qubit fluorometer (Invitrogen) following agarose gel electrophoresis, the PCR products were purified and then quantified. Prior to further processing, an equimolar pool was also obtained. Granted that, the amplicon pool was utilized for pyrosequencing on an Illumina Miseq machine (Macrogen) based on the manufacturer’s protocol.

#### Bioinformatics and data analysis

Consistent with the Illumina processing pipeline, raw reads were filtered initially. The sequences were then analyzed by employing QIIME 1.6.0 software. Besides, the raw reads were also demultiplexed and subsequently filtered through the split_library.py script of QIIME. The analysis was also carried out for 16S rRNA gene reads as follows: the sequences passing the quality filter were denoised and the singletons were removed. It is important to bear in mind that using the uclust method, operational taxonomic units (OTUs) were picked as defined by 97% similarity. Thereafter, the representative sequences—as the most abundant ones in each cluster—were submitted to the RDPII classifier using the Greengenes 16S rRNA gene database to acquire the taxonomy assignment and the relative abundance of each OTU. To finish, QIIME was employed to evaluate alpha and beta diversity.

### Statistical analysis

Next was to use mean and standard deviation (SD) to present the values. Moreover, one-way analysis of variance (ANOVA) and post hoc Tukey’s test was performed using the SPSS Statistics software (version 23). The Pearson correlation coefficient test was also used to examine the correlations between two variables. Statistically, *P *< 0.05 was considered as the level of significance.

## Results

### Levels of oxidative stress markers in the hippocampus and PFC

**Fig. 1:**
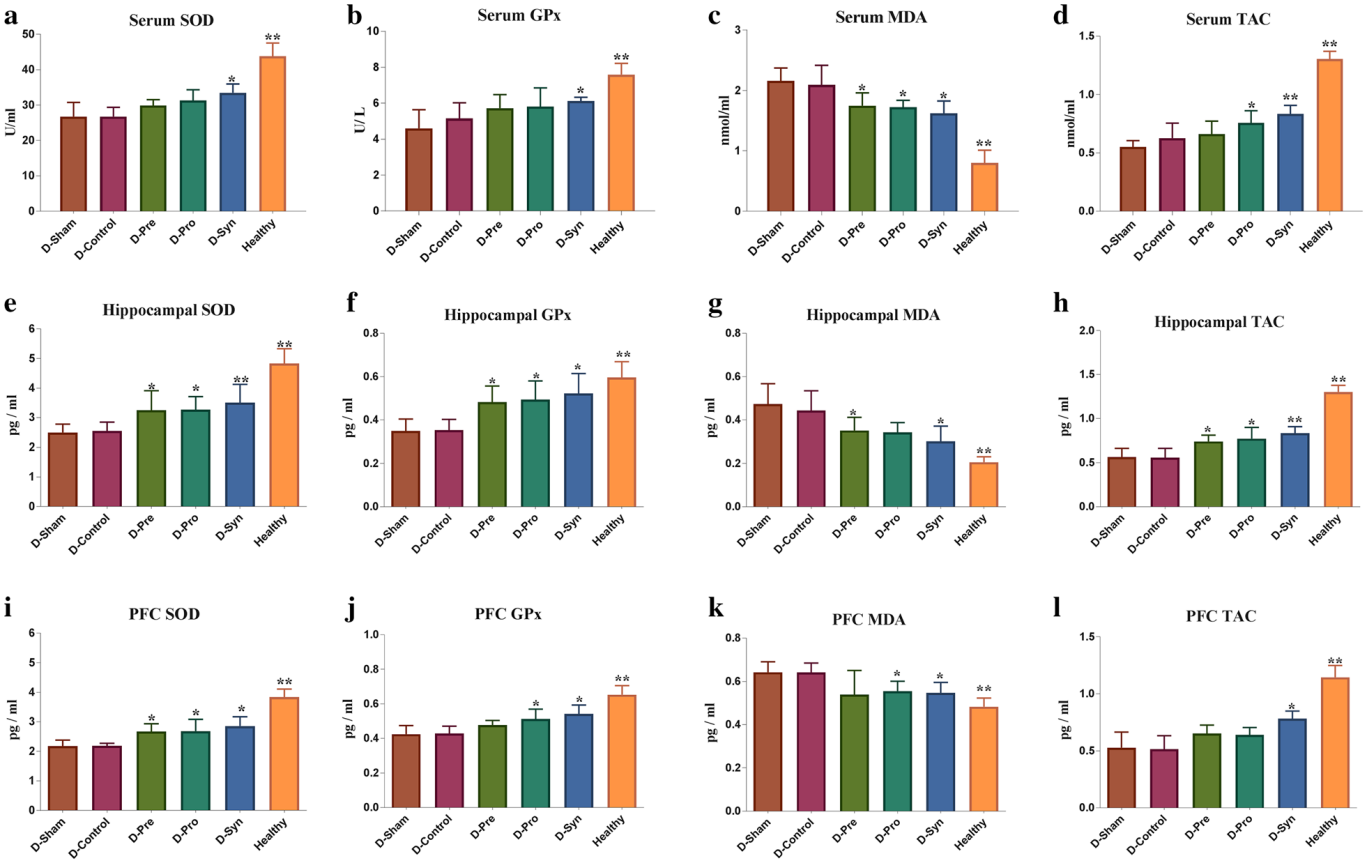
Effects of the *L. plantarum* and inulin on the levels of oxidative stress markers in the blood, hippocampus and PFC of the control and diabetic rats (*n * = 8 per group). **a**–**l** the concentration of the blood, PFC and hippocampal SOD, GPx, MDA, and TAC. One-way ANOVA with post hoc Tukey’s test was used. Data were expressed as means±SD. *P* < 0.05 was regarded as statistically significant. *D-Control* diabetic control, *D-Pro* diabetic + the *L. plantarum*, *D-Pre* diabetic + inulin, *D-Syn* diabetic the *L. plantarum* + inulin, *D-Sham* diabetic sham group

Interestingly enough, the diabetes induction led to increased oxidative stress and decreased levels of antioxidants enzymes (Fig. 1). The results displayed that the blood levels of SOD, GPx, and TAC in the D-Pro and D-Syn groups significantly increased set against the D-Sham group. Besides that, probiotic administration was able to increase levels of TAC. In relation to the D-Sham group, the level of MDA was significantly decreased in all intervention groups. It could be stressed that in both hippocampus and PFC—which is in contrast with the healthy control (Healthy group) rats (P < 0.001)—SOD, GPx, and TAC increased, whereas MDA faced a major decrease in the diabetic sham (D-Sham) group. Inulin supplementation in the diabetic rats resulted in significant elevation of SOD in the hippocampus and PFC (P = 0.020 and P = 0.011, respectively), contrasting with the D-Sham group. Then again, the increase of TAC (P = 0.034) and decrease of MDA (P = 0.037) occurred only in the hippocampus (not in the PFC). By contrast, the GPx levels were not significantly increased in either tissue. The administration of *L. plantarum* ATCC 8014 increased levels of SOD (P = 0.024, P = 0.046), GPx (P = 0.009, P = 0.012), and TAC (P = 0.007, P = 0.24) in the hippocampus and PFC, respectively. In like manner, MDA was decreased (P = 0.009, P = 0.010) in the both tissues, following the supplementation. Moreover, synbiotic administration significantly raised the SOD (P = 0.001, P = 0.020), GPx (P = 0.001, P = 0.020), and TAC (P < 0.001, P = 0.001) levels and decreased MDA concentration (P = 0.001, P = 0.004) in the hippocampus and PFC of the diabetic rats (Fig. 1).

### Serotonin levels in the hippocampus and PFC

**Fig. 2:**
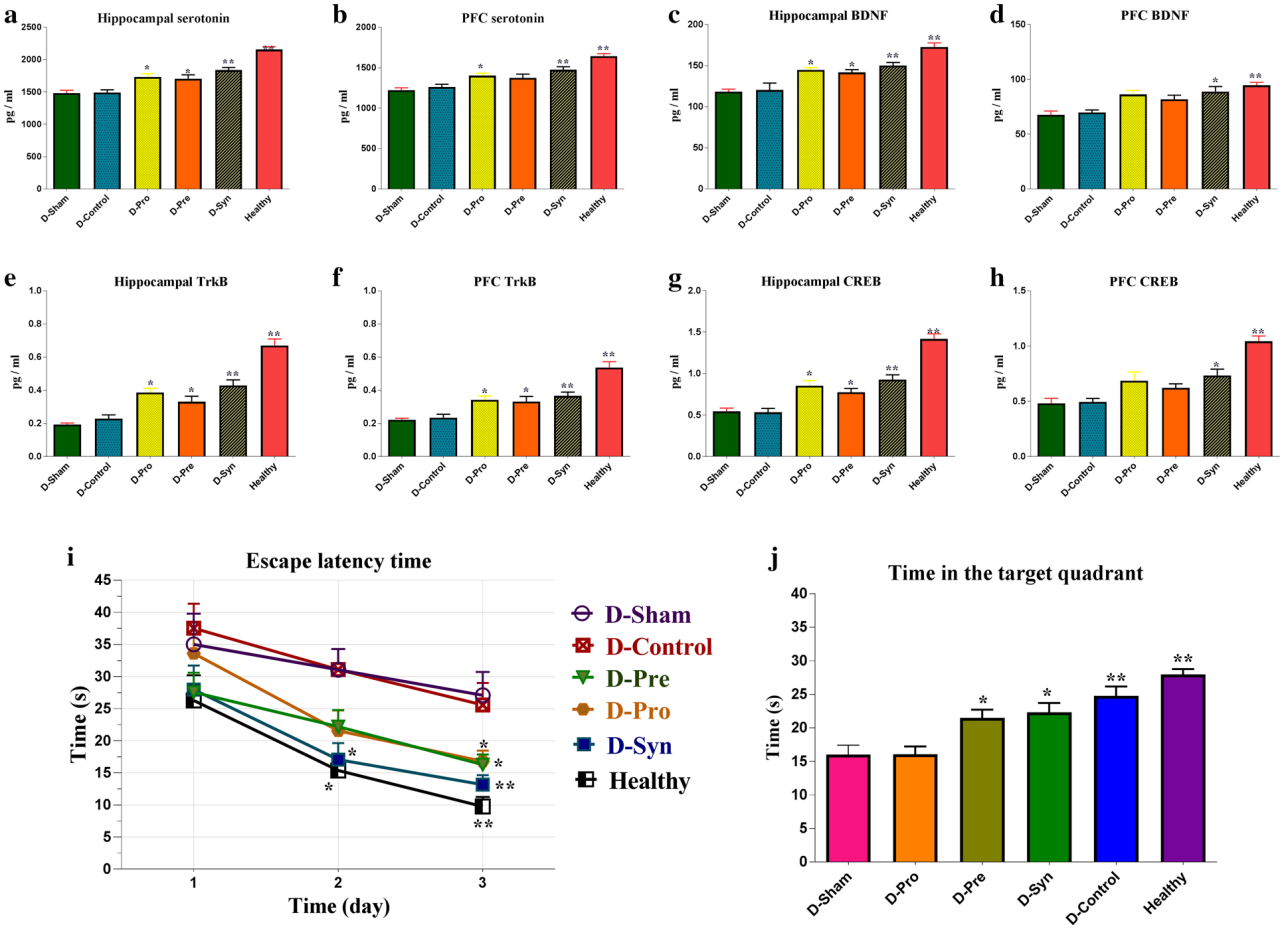
**a**–**h** Alterations of serotonin, BDNF, CREB, and TrkB concentrations in the PFC, hippocampus following supplementation. (*n *= 8 per group). **i** and **j** Effect of the *L. plantarum* and inulin on learning and memory in the Morris water maze test. One-way ANOVA with post hoc Tukey’s test was used. Data were expressed as mean ± SD. *P *< 0.05 was regarded as statistically significant. *D-Control* diabetic control, *D-Pro* diabetic + the *L. plantarum*, *D-Pre* diabetic + inulin, *D-Syn* diabetic the *L. plantarum* + inulin, and *D-Sham* diabetic Sham group, *BDNF* brain-derived neurotrophic factor, *TrkB* tropomyosin receptor kinase B; *CREB* cyclic adenosine monophosphate responsive element-binding protein

The reductions in serotonin levels of the hippocampus and PFC were observed in the diabetic rats (D-Control) through a comparison with the Health group (P < 0.001). In contrast to the D-Sham group (P < 0.001), the usage of the synbiotic in the diabetic rats could increase serotonin level in both tissues. Quaintly, the serotonin concentration in the PFC of diabetic treated with *L. plantarum* + inulin (D-Syn) group could reach the healthy group but no significant difference was detected. In the diabetic group treated with *L. plantarum* (D-Pro), the level of serotonin in the hippocampus (P = 0.010) and PFC (P = 0.047) was higher than the sham group. And to come to the point, such increase (P = 0.024) was observed only in the hippocampus (not in the PFC) of the diabetic group treated with inulin (D-Pre) (Fig. 2).

### BDNF levels in the hippocampus and PFC

Collated with the Healthy group, there was a significant decrease in BDNF level of the diabetic rats (P < 0.001). This reduction was almost reversible by synbiotic therapy in the hippocampus (P < 0.001) and PFC (P = 0.002). Enticingly, increased levels of this neurotrophic factor were noticed in the D-Pro and D-Pre-groups of both Hippocampus (P = 0.004, P = 0.026) and PFC (P = 0.003, P = 0.003), differing from the D-Sham group (Fig. 2).

### CREB Levels in the Hippocampus and PFC

The decreased level of CREB in the diabetic rats was significantly higher than the healthy group (P < 0.001). That said, CREB level increased significantly in the hippocampus of the diabetic rats, supplemented with *L. plantarum* (P = 0.001) and inulin (P = 0.046) and their combination (P < 0.001), compared to the sham group. Yet in the PFC tissue, the elevation of CREB level was only observed in the synbiotic group (P = 0.025), which is in contrast with the D-Sham group and was not significant in the two other intervention groups (Fig. 2).

### TrkB levels in the hippocampus and PFC

It is critical to state that the level of TrkB in diabetes status expressively decreased in the D-Sham group (P < 0.001) which was in contrast to the healthy group. Despite this, there was a significant increase in the levels of this receptor in the hippocampus and PFC in all three intervention groups, which is different from that of the D-Sham rats. And on top of that, the level of TrkB in the hippocampus and PFC in the D-Pro (P = 0.001, P = 0.013), D-Pre (P = 0.028, P = 0.039), and D-Syn (P < 0.001, P = 0.004) groups were significantly higher than the D-Sham group (Fig. 2).

### Effect of supplements on learning and memory

Learning and memory of the rats were measured using the Morris water maze (MWM) test (Fig. 2i). On the first day of the test, there was no significant difference in the time of finding the hidden platform among the rats. With that in mind, the healthy rats revealed better performance than the D-Control group in the upcoming days (second day; P = 0.003 and third day; P < 0.001). Also, on the second day, the mean latency in finding the platform in the rats treated with synbiotic was much higher than that of the D-Sham group (P = 0.011). Nevertheless, there were no significant differences in the time of finding the platform between other interventions and D-Sham group on the second day. On the third day, the supplementations enhanced the process of finding the platform in each of the three intervention groups. The escape latency in the D-Pre (P = 0.031), D-Pro (P = 0.002), and D-Syn (P < 0.001) rats was less than the D-Sham group.

In the probe trial, the long-term memories of the experimental groups were measured (Fig. 2j). At this step, the time spent in the target quadrant was significantly more in the diabetic rats supplemented with *L. plantarum* (P = 0.013), inulin (P = 0.040), and their combinations (P < 0.001), vis-à-vis the D-Sham group. The time spent on the target quadrant in the D-Syn group was very close to the Healthy group. Unlike other groups, no significant difference was observed between the D-Syn and Healthy group. Provided that, the D-Control rats had a lower elapsed time in this quarter, putting together with the Healthy group (P < 0.001), indicating impaired memory in the diabetic rats.

### Alterations of the gut microbiome

**Fig. 3:**
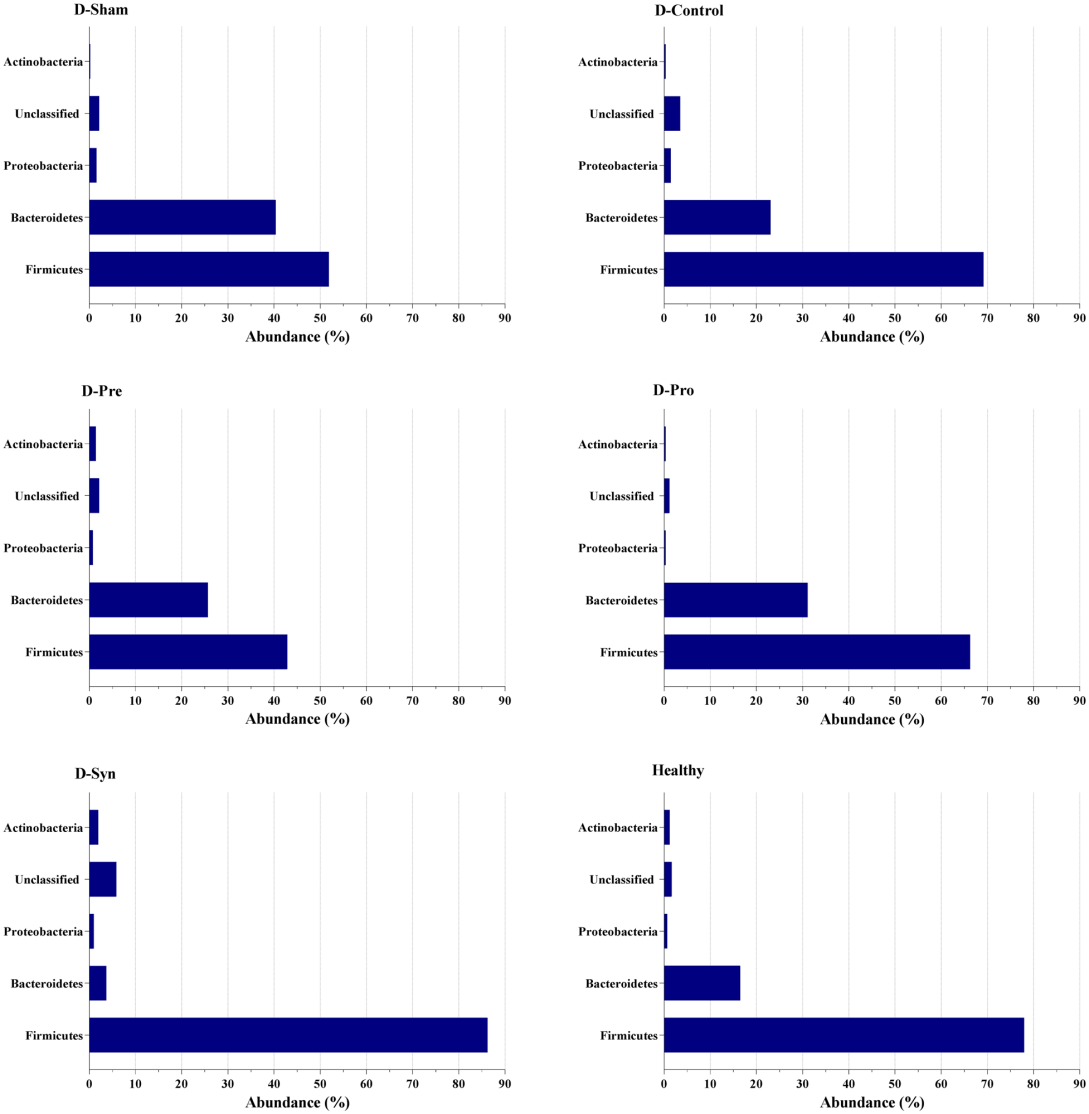
Effect of the *L. plantarum* and inulin on the taxonomic profiles (Phyla) of fecal microbiota (*n *= 8 per group). One-way analysis of variance, followed by post hoc Tukey’s test, was used. Data were expressed as means±SD. *P * < 0.05 was regarded as statistically significant. *D-Control* diabetic control, *D-Pro* diabetic + the *L. plantarum*, *D-Pre* diabetic + inulin; *D-Syn* diabetic the *L. plantarum* + inulin, *D-Sham*: diabetic Sham group

**Fig. 4:**
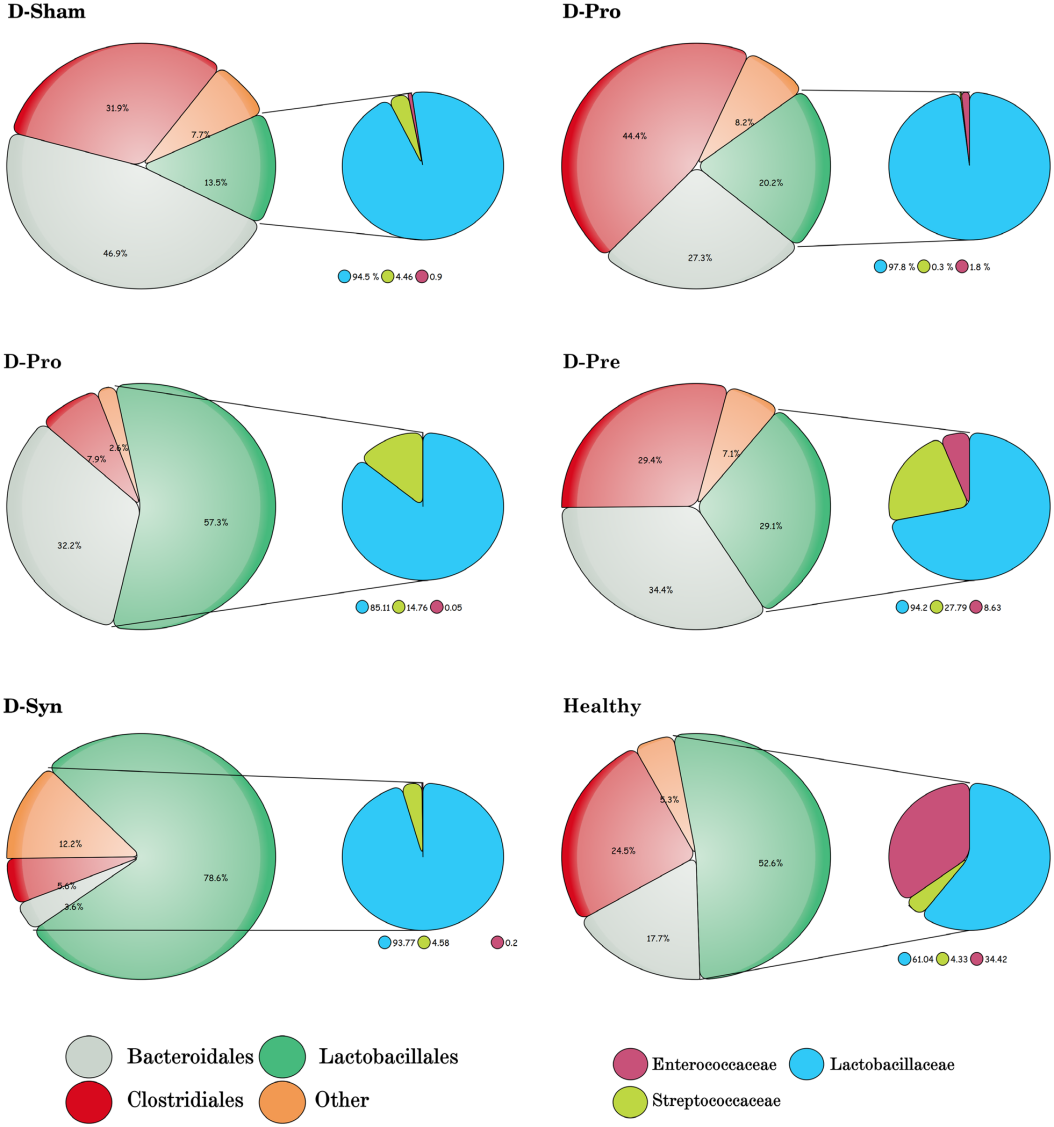
The effect of the *L. plantarum*, inulin, and their concurrent administration on the composition and diversity of the gut microbiota (Order and Family assignment). ANOVA analysis with post hoc Tukey’s test were performed to identify statistically significant differences. Data were expressed as relative abundance. *D-Control* diabetic control, *D-Pro* diabetic + the *L. plantarum*, *D-Pre* diabetic + inulin, *D-Syn* diabetic the *L. plantarum* + inulin, *D*-*Sham* diabetic sham group

**Fig. 5:**
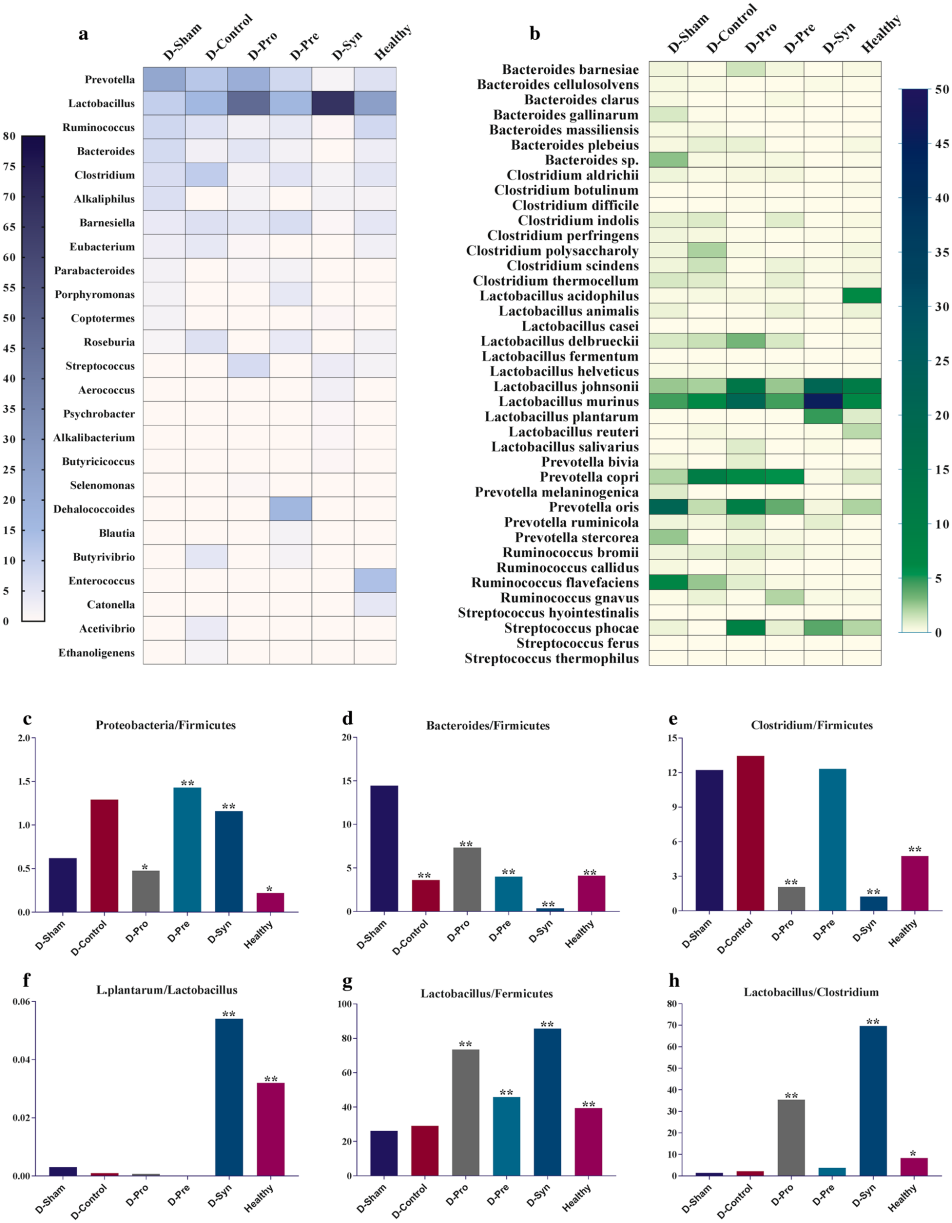
**a**, **b** The Heat map show the taxonomic abundances of most repeated species and strains (OTUs). **c**–**h** The ratios based on metagenomics data in fecal samples (phyla). *D*-*Control* diabetic control, *D*-*Pro* diabetic + the *L. plantarum*, *D-Pre* diabetic + inulin, *D*-*Syn* diabetic the *L. plantarum* + inulin, *D*-*Sham* diabetic sham group

The taxonomic profiles of fecal microbiota outlined remarkable changes in the gut bacterial composition and population of the diabetic vs. healthy rats. In like fashion, the composition of the microorganisms (Phylum) of each group specified a significant increase in the population of *Firmicutes* bacteria in the treated groups (D-Syn and D-Pro), collating with the D-Sham rats (Fig. 3). It was also demonstrated that the abundance of the *Bacteroidetes* was raised more than 35% in the D-Sham, compared to the D-Syn, D-Pro, D-Pre, and Healthy group groups (Fig. 3). Strikingly, the evaluation of Order-based bacterial groups and their sub-groups (Family) manifested that the relative abundance of *Lactobacillales* significantly increased while *Clostridiales* and *Bacteroidales* bacteria dramatically reduced in the D-Syn and D-Pro groups. In contrast to other treated groups, prebiotic (D-Pre) consumption had the utmost effect on increasing bacterial population in the *Streptococcaceae* classification of the *Lactobacillales* members. Interestingly enough, another factor was the significant reduction of the highest population of *Enterococcaceae* family by complementarity in groups D-Pro and D-Syn which was reported in the healthy group (Fig. 4). Furthermore, 40 bacterial species of high significance were reported. It is absorbing to note that *Lactobacillus* represented the highest percentages of the population in the treated groups (D-Pro and D-Syn), while the dominant Genera in the D-Sham group were *Prevotella*, *Ruminococcus*, and *Clostridium* (Fig. 5a). Also, the Probiotic and synbiotic intake could significantly increase the abundance of *Lactobacillus* species in the genera classified. As it is visible in the Fig. 5b, *L. delbrueckii*, *L. murinus* and *L. johnsonii* were the species with the highest frequency detection in the D-Syn and D-Pro groups while in the D-Sham group, *P. oris* and *R. flavefaciens* were the most prevalent bacterial species. The change of the composition and population helped in the clarification of the point (Fig. 5b). It should be pointed out that the significant increases in the ratio of *Lactobacillus/Fermicutes* were witnessed in all treated groups (D-Syn, D-Pro, and D-Pro). Another key thing to remember is that a significant difference was spotted only in the D-Syn and D-Pro groups setting the mentioned matter against the proportion of *Lactobacillus/Clostridium* (Fig. 5). Moreover, the greatest ratio of *Bacteroides/Firmicutes* (14.4%) and *Clostridium/Firmicutes* (12.2%) were observed in the diabetic rats reversed by supplementation, especially synbiotic intake (Fig. 5d, e). One remarkable observation in our metagenomics analyses was the dramatic increase in the *L. plantarum* population only in the D-Syn group (Fig. 5f).

## Discussion

This study set out with the aim of assessing the importance of gut microbiota alteration in neuropsychological markers in diabetic rats. The current study found that *L. plantarum* ATCC 8014 and inulin could reverse the cognitive impairment of the diabetic rats in the current work. Following the supplementation, improved antioxidant capacity was similarly observed in the hippocampus and PFC. In this respect, normalization of the gut microbiota after the intervention could increase the concentration of serotonin and BDNF and enhance the BDNF/TrkB/CREB pathway in the two brain regions. It was also found that the concurrent intake of *L*. *plantarum* and inulin performed a synergistic effect.

To continue, the evidence was demonstrating of the fact that gut microbiota plays a noteworthy role in the development and functioning of the CNS [12, 32]. Precise pathways or mechanisms between the gut and brain remain nonetheless unclear. In the present study, the ability of probiotics and prebiotics to make favorable changes in the gut microbial composition was demonstrated. Likewise, the findings of this study argued that the supplements are capable of increasing the population of beneficial bacteria. It was also discovered that *L. plantarum*, set against inulin, plays a rather effective role in raising several *Lactobacillus* species and reducing the *Clostridium* bacteria population. It is noteworthy to state that the study synbiotics, compared to separate supplementation, had a stronger role in reducing *Clostridium* and *Bacteroides* as well as increasing *Lactobacillus* population. It is newsworthy to state that there is a controversy regarding the synergistic role of probiotics and prebiotics. Keshavarzian et al. [33] illuminated that separate intake of galactooligosaccharide (GOS) and *B. lactis* BB-12 for 3 weeks led to a significant reduction of anxiety in obese mice, while such amelioration was not observed in their concurrent use. According to the present investigation, inulin caused no major changes in the growth of probiotics such as *Bifidobacterium*, whereas co-administration of inulin and *L. plantarum* increased the population of *L. plantarum* and other potentially beneficial microbes such as *Streptococcus*. These findings stated that the type of prebiotic is a key factor in determining the growth of a probiotic bacterium. Furthermore, the duration of the intervention with inulin is crucial and may require a longer intervention period to exert a significant effect on the growth of probiotics [34]. The focal point was that *L. plantarum* administration alone did not increase the gut *L. plantarum* population but it reduced the growth of *Clostridium* and increased other *Lactobacillus* species. The above finding suggests that taking a probiotic species does not necessarily lead to an increase in the population of the same species. It is noteworthy to highlight that not only the evaluation of overall changes in the gut microbiota such as the total population of *Lactobacillus* are to be noted; however, changes in various species following the supplementation are also considerable. Although supplementation with probiotics (D-Pro) could not increase the growth of *L. plantarum* population, it lead to a dramatic increase in the population of other similar bacteria such as *L. morinos*, *L. delbrueckii* and *L. acidophilus* which have anti-oxidative, anti-bacterial and anti-diabetic effects [35–38]. In this regard, Ejtahed et al. [37] elucidated that supplementation with *L. acidophilus* could decrease the levels of LDL-C, total cholesterol, FBS, and increase HDL-C concentration in type 2 diabetic patients. According to the reports, the ratio of different bacterial groups to one another may even be more distinguished pitting against their populations. Overall, by increasing the proportion of beneficial species toward harmful species, more effective results could be achieved. To illustrate, improvement of the key bacterial ratios such as *Lactobacillus/Clostridium* after supplementation was observed, specifically after the symbiotic intake. Along with our study, Larsen et al. [39] disclosed that the gut microbiota in human adults with T2DM differs from non-diabetic adults. In their study, the proportions of phylum *Firmicutes* and class *Clostridia* were significantly reduced in diabetic patients. By and large, positive correlations could also be noted between the ratios of *Bacteroidetes* to *Firmicutes* as well as the ratios of the *Bacteroides/Prevotella* group to plasma glucose concentration.

**Fig. 6:**
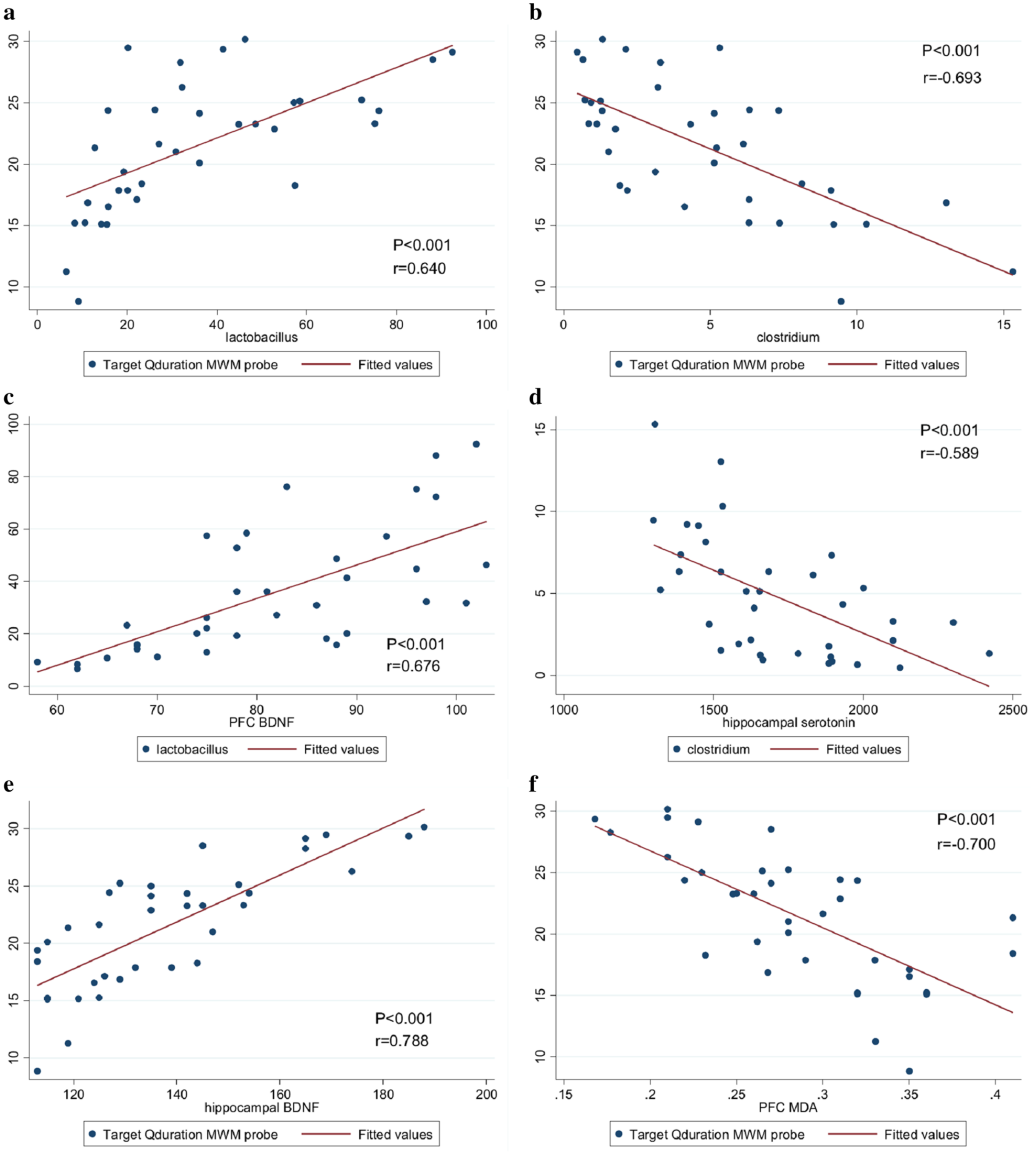
Correlation coefficients between the gut microbiome (*Lactobacillus* and *Clostridium*) relative abundance with learning and memory in Morris water maze. Correlation of **a***Lactobacillus*, **b***Clostridium* with time in the target quadrant, **c** PFC BDNF concentration with lactobacillus, **d** Hippocampal serotonin concentration with *Clostridium*. **e** Hippocampal BDNF level with time in the target quadrant. **f** PFC MDA level with time in the target quadrant. Relations between two variables were computed by Pearson correlation coefficients. *D*-*Control* diabetic control, *D-Pro*: diabetic + the *L. plantarum*, *D*-*Pre* diabetic + inulin, *D*-*Syn* diabetic the *L. plantarum* + inulin, *D*-*Sham*: diabetic sham group, *BDNF* brain-derived neurotrophic factor, *TrkB* tropomyosin receptor kinase B, *CREB* cyclic adenosine monophosphate responsive element-binding protein

It is to be pointed out that the induction of T2DM resulted in the elevation of oxidative stress markers in the two brain regions. In the present study, it was demonstrated that the inulin and *L. plantarum* had the potential to improve oxidative status; however, this enhancement was more impressive after the synbiotic intake. SOD and MDA levels had the most noticeable changes, following the supplementation. It was also reported that the administration of the supplements could prevent severe weight loss and reduce polyphagia, blood glucose levels and IR (Additional file 1: Fig. S1A, B). Furthermore, the treated groups presented a substantial reduction of blood glucose and increased insulin sensitivity (Additional file 1: Fig. S1C, D). Intriguingly, oxidative stress is one of the most considerable indicators of T2DM, which leads to a wide range of complications [40]. Moreover, hyperglycemia and IR intensify lipid and protein peroxidation and hence functional and structural damage to the nerve [41]. The improvement of antioxidant capacity can be considered as a contributing factor in the prevention and treatment of neurological disorders [42]. On the same subject, Chen et al. [43] reported in a study that intake of *L. casei* CCFM0412 (10^9^ CFU/day) for 12 weeks resulted in elevated serum GSH, SOD, GPx concentrations and reduced the levels of ROS and MDA in mice with T2DM. Kleniewska also revealed that co-administration of inulin (400 mg) and *L. casei* (10^8^ CFU/mL) for 7 weeks increased serum levels of SOD, catalase, and GPx in healthy subjects [44]. On the other hand, Kapczinski demonstrated that increasing oxidative stress led to a decrease in BDNF concentration in bipolar disorder [45]. In their study, an improvement of oxidative stress was reported in lipoic acid-treated rats, following improvement of the gut microbiota. A positive correlation of ROS and MDA with *E. coli* and *Enterococcus* as well as TAC with lactobacilli was observed withal [46]. Likewise, the present study demonstrated a strong correlation between the *Lactobacillus* and SOD as well as MDA levels in the hippocampus and PFC of the diabetic rats (Fig. 6).

One of the most revealing findings on the research in hand was the increase in serotonin concentration as well as the strengthening of the BDNF/TrkB/CREB pathway due to the interaction with the supplements. BDNF and serotonin play a pivotal role in maintaining and improving neuropsychological functions and preventing nerve damage [18]. Moreover, these two parameters with synergistic function play a key role in the mechanisms of cognitive behavior [18]. Interestingly enough, reducing the level of either parameter can trigger a decrease in the other parameter and vice versa. On a similar note, Borrelli et al. [47] indicated that *L. rhamnosus* administration led to increased brain expression levels of BDNF and serotonin as well as raised fecal *Firmicutes* and reduced *Proteobacteria* population in zebrafish . Evidence has presented that it requires two noteworthy receptors, i.e. TrkB and CREB, to be activated as well for the effectiveness of BDNF [47, 48]. TrkB is a specialized receptor of BDNF which decrease is observed in behavioral disorders [49]. Correspondingly, Guoyuan et al. [50] illustrated in their study that tea polyphenols had protective effects against oxidative stress-triggered cognitive impairment via modulation of the BDNF/TrkB/CREB signaling pathway in housing mice in constant darkness. Regarding the same, Xiang et al. [51] also found that improvement of this pathway in the brain could improve memory impairment in Alzheimer’s mouse models. In this respect, it was illustrated that there was a strong correlation between the levels of brain parameters and the population of *Lactobacillus* and *Clostridium* bacteria (Fig. 6). It is worth mentioning that Synbiotic administration was associated with expressive shifts in all of the study parameters including serotonin, BDNF, TrkB, and CREB, while *L. plantarum* or inulin singlehandedly improved only some of the parameters in the hippocampus and PFC. Such changes were related to a normalized gut microbial composition and improved state of the oxidative stress which prevented neuronal damage and proteins oxidation. Consequently, the use of supplements could increase serotonin levels and ameliorate TrkB/BDNF/CREB pathway via increasing the ratio of *Lactobacillus*/*Clostridium* and *Lactobacillus*/*Bacteroides*, in addition to enhancing antioxidant capacity.

In the present study, it was explored that supplements intake could significantly enhance the T2DM induced cognitive impairment. Moran et al. [52] suggested that brain atrophy and cognitive impairment are increased in T2DM patients, collated with the control group. Alternatively, Burokas et al. [53] disclosed that administration of GOS and FOS resulted in improved anxiety and depression by normalizing the gut microbiota in mice with chronic stress. In a recent study conducted by Chunchai et al. [34], consumption of probiotic (*L. paracasei* HII01), prebiotic (xylooligosaccharide), and synbiotic for 12 weeks led to the amelioration of memory and learning as well as hippocampal plasticity and attenuation of the hippocampal oxidative stress in HFD-treated rats. Mentionable it is that in the present work, it was also elucidated that there is a strong correlation of memory with serotonin and BDNF in the hippocampus and PFC (Fig. 6). Given this orientation, the close association of SOD and MDA levels with memory was noticed.

One of the interesting results obtained in the study in hand was the correlation between common gut bacteria and memory (Fig. 6). The data in this study revealed that the increase in the *Lactobacillus* and decrease in the *Clostridium* population were highly correlated with learning. Fascinatingly, the highest correlation was perceived between *Lactobacillus*, *Bacteroides*, and *Clostridium* and bacteria population. It should be mentioned that there was a strong correlation between IR, blood glaucous, weight gain with *Lactobacillus* and *Clostridium* abundance. In a study conducted by Kang et al. [54] it was presented that relative abundance of *Streptococcus* was more observed in HFD-treated rats and was accompanied with increased anxiety-like behavior. For this reason, the reduction of the population of *Clostridium* and *Bacteroides* can prevent cognitive impairment, following the use of the supplements, especially synbiotics. Then again, studies in this field are still limited and there is no clear mechanism for the gut–brain interaction in improving behavioral disorders. Consequently, there were some limitations in this work; perhaps one of the most striking limitation was that the effect of the supplements on the gut microbial composition at weekly intervals over a period of 8 weeks could not be evaluated.

## Conclusions

In general, the results of the present study were suggestive of the favorable effects of supplements on improving the gut microbial composition. The administration of synbiotics had a major excessive role versus mere inulin or *L. plantarum*. Added to that, the results implied that the supplements had the ability to increase antioxidant capacity. What is more, the protective and strengthening effects of the supplements were observed on serotonin concentration and BDNF/TrkB/CREB pathway in the hippocampus and PFC. To conclude, the improvement of the gut microbiota composition, oxidative stress, and levels of brain parameters led to ameliorated learning and memory impairment in the diabetic rats. As a final point, manipulating the gut microbiota can be considered as a therapeutic target for the prevention and treatment of metabolic and neuropsychological disorders. Due to the highly limited researches in this field, further studies are warranted.

## Supplementary information

**Additional file 1: Fig. S1**.

## Data Availability

All data are available in the manuscript or upon request to the authors.

## Abbreviations

BDNF: brain-derived neurotrophic factor
CREB: cyclic adenosine monophosphate responsive element-binding protein
GPx: glutathione peroxidase
HFD: high fat diet
IR: insulin resistance
LPS: lipopolysaccharides
MDA: malondialdehyde
MWM: morris water maze
PFC: prefrontal cortex
SOD: superoxide dismutase
STZ: streptozotocin
TAC: total antioxidant capacity
TrkB: tropomyosin receptor kinase B
T2DM: type 2 diabetes mellitus
