# Supplementary figures and images for: The potential therapeutic effects of the gut microbiome manipulation by synbiotic containing-Lactobacillus plantarum on neuropsychological performance of diabetic rats
Source: J Transl Med. 2020 Jan 10;18:18. doi: 10.1186/s12967-019-02169-y (PMC6953298; doi:10.1186/s12967-019-02169-y)

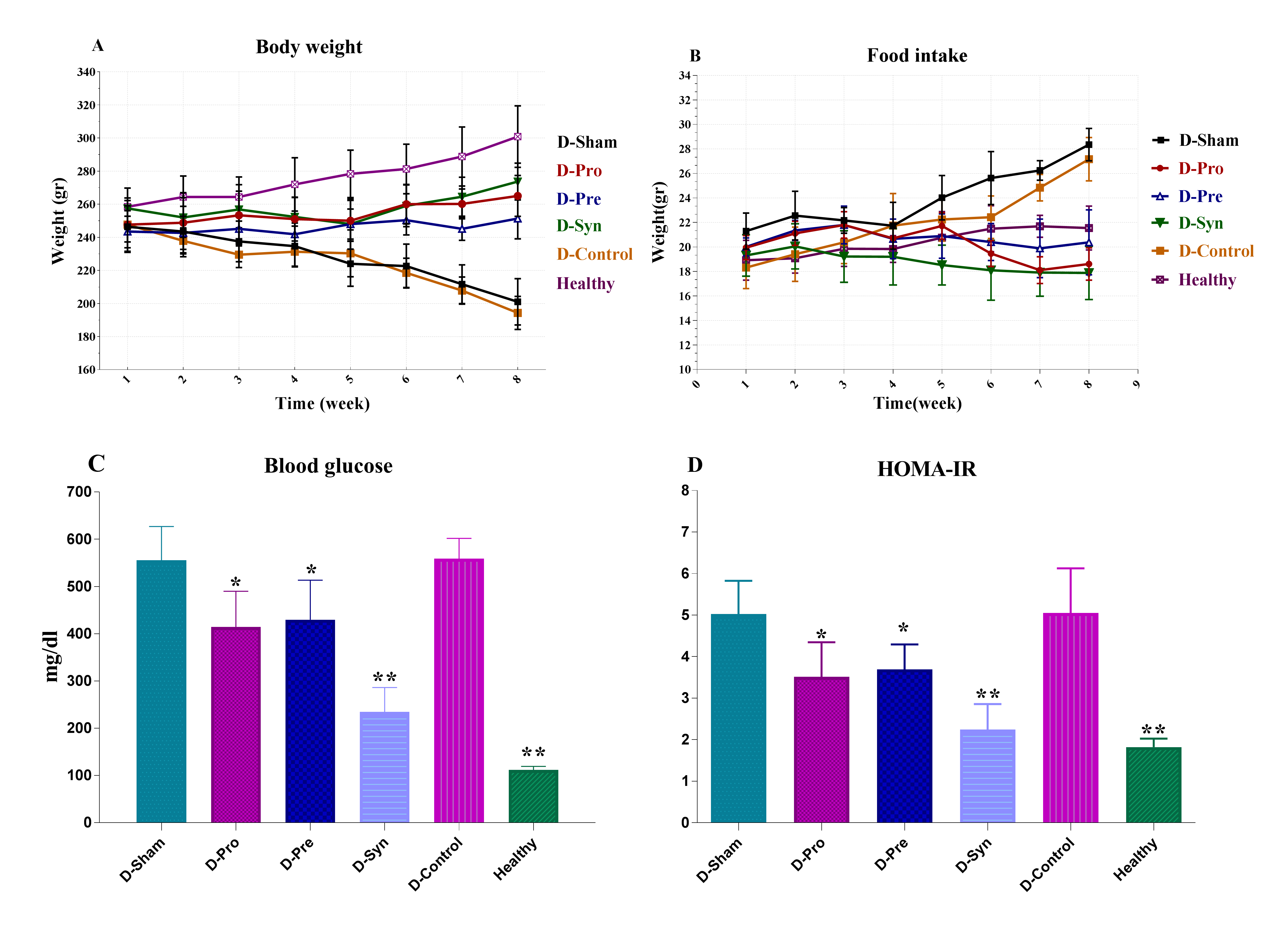

Supplement: Supplementary file 1 — Additional file 1: Fig. S1. [file 12967_2019_2169_MOESM1_ESM.tif]
